# Detoxification and decolorization of complex textile effluent in an enzyme membrane reactor: batch and continuous studies

**DOI:** 10.3389/fmicb.2023.1193875

**Published:** 2023-07-07

**Authors:** Manju Dahiya, Dar Tafazul Islam, Preeti Srivastava, T. R. Sreekrishnan, Saroj Mishra

**Affiliations:** Department of Biochemical Engineering and Biotechnology, Indian Institute of Technology Delhi, New Delhi, India

**Keywords:** continuous treatment, enzyme membrane reactor, engineered laccase, real textile effluent, recombinant laccase

## Abstract

There is an urgent need to look for bio-based technologies to address the pollution related to textile dyes in waterbodies. The aim of this study was to evaluate an engineered laccase variant, LCC1-62 of *Cyathus bulleri*, expressed in recombinant *Pichia pastoris*, for the decolorization and detoxification of real textile effluent. The partially purified laccase effectively (~60–100%) decolorized combined effluent from different dyeing units at a laccase concentration of 500 U/L at a 50-mL level. Decolorization and detoxification of the combined effluents, from a local textile mill, were evaluated at 0.3 L volumetric level in a ray-flow membrane reactor in batch and continuous modes of operation. In batch studies, maximum decolorization of 97% and detoxification of 96% occurred at a hydraulic retention time (HRT) of 6 h without any additional laccase requirement. In continuous studies, the reactor was operated at an HRT of 6 h with a lower enzyme dosage (~120 U/L of the effluent). Decolorization was accompanied by a loss in laccase activity which was restored to ~120 U/L by the addition of laccase in two regimes. The addition of laccase, when the residual laccase activity decreased to 40% (~50 U/L), resulted in high decolorization (~5 ppm residual dye concentration) and low variance (σ^2^) of 2.77, while laccase addition, when the residual dye concentration decreased to ~8% (~10 U/L), resulted in an average dye concentration of 13 ppm with a high variance of 62.08. The first regime was implemented, and the continuous reactor was operated for over 80 h at an HRT of 3 and 6 h, with the latter resulting in ~95% decolorization and 96% reduction in the mutagenicity of the effluent. Less than 10% membrane fouling was observed over long operations of the reactor. The findings strongly suggest the feasibility of using LCC1-62 in an enzyme membrane reactor for large-scale treatment of textile effluents.

## Introduction

Laccases have a high potential in the textile industry, both in the free and immobilized forms (Chatha et al., 2017; Zdarta et al., 2021), for decolorization, degradation, and detoxification of dyes and can be efficient at the industrial scale. These enzymes have also been used successfully for the removal of toxic phenolic contaminants (Mukherjee et al., 2013) generated from other industries. The effluents from the textile industries, containing dyes and other auxiliaries (such as detergents, heavy metals, salts, and starch) pollute both small and large waterbodies leading to coloration, toxicity, and carcinogenicity. Apart from this, the dyes and other additives also lead to changes in pH, an increase in the biological and chemical oxygen demand (BOD and COD), and other particulate matter. Annually, approximately 7 × 10^7^ tons of synthetic dyestuff are produced with approximately 10,000 tons being used in the textile industries (Chandanshive et al., 2020). The currently available methods for textile effluent include physico-chemical treatment followed by aerobic processes that achieve a reduction in BOD as well as in COD but are accompanied by the generation of large amounts of sludge. Although several advanced oxidation methods, electrochemical, and photo-electrocatalytic treatments are available, these are expensive to implement at large scale (Azanaw et al., 2022), and hence biological methods are preferred. The white-rot fungi in free and immobilized form have been used for the decolorization and degradation of several model dyes and dye-containing real effluent (Chakraborty et al., 2013; Spina et al., 2014; Mir-Tutusaus et al., 2018). While this method can be used for shorter durations of treatment, these are not effective over longer periods as the fungal growth is compromised.

Enzymes such as azo reductases and those of lignin degradation, such as laccases, lignin peroxidases, manganese peroxidases, and versatile peroxidases (Singh et al., 2021) have been shown to be effective in removing lignin derivatives, phenolics, and color from the textile effluents. However, the use of azo reductases results in the formation of arylamines, while the synthetic mediators used with laccases are poisonous and lead to an increase in toxicity (Vats and Mishra, 2017, 2018). Several studies report the development of catalytically efficient laccases (Stanzione et al., 2020) that show tolerance to high pH, temperature, metal ions, and salts, properties useful for application in the textile industry. For instance, laccases with superior catalytic efficiency on high-redox/complex dyes (Kenzom et al., 2014) have been developed. Similarly, thermally stable (Mateljak et al., 2019) and alkali tolerant (Yin et al., 2019) laccases have been developed through random (followed by using a strong screen for the specific feature) or site-directed (predictions based on molecular modeling, quantum mechanics, and molecular dynamics simulations) mutagenesis (Mehra et al., 2018). In addition to this, screening through directed evolution, assays of chimeric structures, and synthetic biology have also led to mutants that displayed stability toward high temperature, chemical inhibitors, and organic solvents and displayed alternately directed substrate specificities and increase in the standard redox potential at the T1 site (Mateljak et al., 2019). The T1 site is the substrate binding site in the laccases where the oxidation of the substrate occurs. Structurally, such mutations involved the modification of functional groups at the substrate binding site, coordination at the T1 copper site, and introduction of stabilizing mutations in the interfaces of various laccase domains (Herrera-Zúñiga et al., 2019).

For large-scale treatment of textile effluent, effluent containing phenolic compounds, and emerging contaminants (pharmaceuticals), the use of enzymatic membrane reactors (EMRs) has been recommended (Calabrò et al., 2009; Chhabra et al., 2009, 2015a; Lloret et al., 2012). While several groups (Chhabra et al., 2015b; Gu et al., 2021) have reported the use of either free or immobilized laccase–mediator system for the treatment of dyes and textile effluent, there are no reports on the use of free laccase for the treatment of dyes. Furthermore, the addition of mediators increases the cost of operation and may increase further the toxicity of the effluent. This is particularly true for synthetic mediators such as 1-hydroxybenzotriazole (HOBT) or ABTS (https://www.sigmaaldrich.com/IN/en/sds/aldrich/54802, https://cdn.caymanchem.com/cdn/msds/27317m.pdf). Thus, there is a need to look for efficient laccases that can be used without the addition of any mediator. In the present study, the engineered laccase variant LCC1-62 (possessing Ile_490_Met substitution with improved K_cat_) of laccase 1 (LCC1) isoform of *Cyathus bulleri* produced in the recombinant *Pichia pastoris* strain was used. The LCC1-62, shown previously to be effective on high-redox dyes (Kenzom et al., 2014), was evaluated in a membrane reactor for its efficiency in the treatment of real effluent. The first set of experiments was performed in batch mode to optimize the operating conditions which were then implemented in a continuous reactor for the treatment of the effluent.

## Materials and methods

### Chemicals and equipment

ABTS [2,2′-azinobis-(3-ethylbenzothiazoline-6-sulfonic acid)] utilized for measuring enzyme activity was purchased from Sigma-Aldrich. Bradford's reagent used for protein estimation was obtained from Fermentas, USA. The membrane separation unit (Ray-flow X100; Orelis, France) was used for enzyme filtration during reactor operations. Ultra-filtration membrane (10 kDa) was purchased from Permionics, India.

### Production and partial purification of laccase variant

Routine maintenance of the recombinant *P. pastoris* clone expressing either the wild-type (WT) LCC1 isoform or the mutant derivative LCC1-62 (Kenzom et al., 2014) was carried out on YPD (1% yeast extract, 2% peptone, and 2% dextrose) plates (containing 2% agar) from a glycerol stock culture (YPD + 15% glycerol) stored at −70°C. For the production of extracellular WTLCC1 or the LCC1-62 laccase, the respective recombinant clones were cultivated in YPD, followed by transfer to 500 mL × 4 flasks containing buffered complex glycerol medium (BMGY) and then to buffered complex methanol medium (BMMY) according to Invitrogen *Pichia* Expression Kit manual (Invitrogen, USA). The culture was induced with methanol (to a final concentration of 1%, v/v) every 24 h for maintaining induction and cultivated at 28°C for 120 h. Extracellular protein concentration, laccase activity, and cell OD600 were monitored every 24 h for 5 days as described under analytical methods. After harvest, the enzyme was partially purified by subjecting the cell-free culture supernatant to vacuum filtration through a 0.45 μm membrane filter followed by ultrafiltration using polyethylene sulfonate membrane (10 kDa) (Millipore, USA) in an ultrafiltration cell (Amicon, USA). The partially purified laccase preparations were kept at −20°C for long-term storage where the activity was retained by over 90% over a period of 6 months.

### Screening of the laccase variant on different textile effluents

The primary screening of LCC1-62 was carried out for the decolorization of five effluents, namely, Eff 1 (dyeing bath effluent, Alps Industries, Meerut, U.P.), Eff 2 (combined effluent from denim dyeing unit 1, Rohtak, Haryana), Eff 3 (combined effluent from denim dyeing unit, Ghaziabad, U.P.), Eff 4 (combined effluent from denim dyeing unit 2, Rohtak, Haryana), Eff 5 (combined effluent-batch 1, from Alps Industries, Meerut, U.P.), and Eff 6 (combined effluent-batch 2, from Alps Industries, Meerut, U.P.). The composition of the effluents is shown in Supplementary Table S1. Since the treatment process was to be designed for the combined effluent from Alps Industries, Eff 5 and Eff 6 were taken for batch and continuous studies, respectively. Eff 5 contained predominantly Disperse Red dye, while Eff 6 contained Disperse Blue dye. The dyes used in the industry were the azo dyes (C.I. dyes, namely, Disperse Yellow 211, Disperse Red 73, and Disperse Blue 173), and the structure of these dyes is shown in Supplementary Figure S1. These provide light fastness and high shade durability. The total concentration in mg/L (ppm) of the dyes in the combined effluent was ~90. This is appropriate as the pH of the combined effluent is generally around 9–10 and needs to be adjusted to 4.0, the optimum for the laccases (Kenzom et al., 2014) and the WTLCC1 and LCC1-62 were added at 500 U/L of the reaction mixture (set in duplicate in 25 mL flasks). The flasks were incubated at 30°C (in the dark) with shaking at 50 rpm till maximum decolorization was achieved. Percent decolorization was calculated by taking the area under the absorption spectrum recorded from 320 to 700 nm. The time of maximum decolorization was noted for all the samples.

### Ray-flow membrane reactor

**Figure 1:**
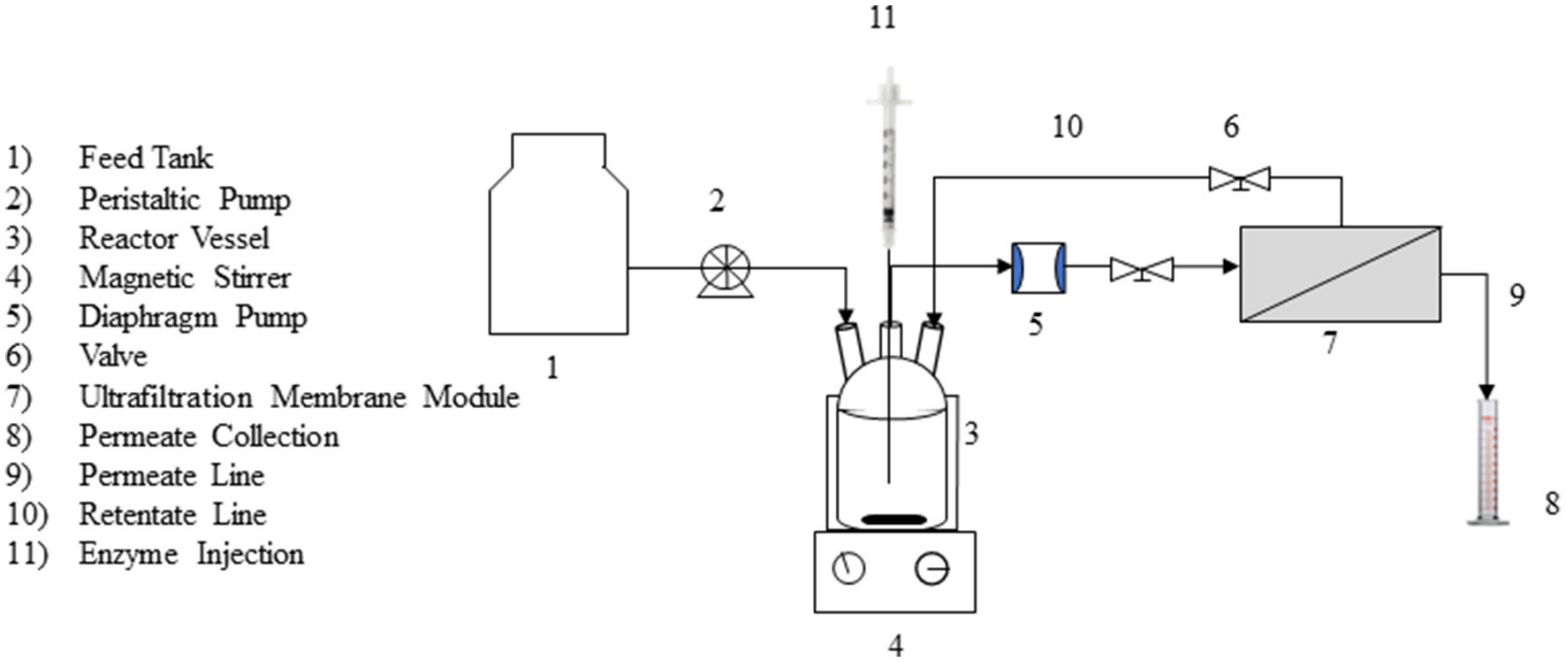
Schematic diagram of the membrane reactor used in the present study.

A lab-scale ray-flow membrane unit (Supplementary Figure S2) was set up with an ultrafiltration poly-sulfone (PS) membrane with a molecular weight cutoff of 10 kDa. The membrane was procured from Permionics Membranes Pvt. Ltd. (Vadodara, Gujrat). The layout of the experimental set-up in the continuous mode is shown in Figure 1. The set-up consisted of a 300-mL ray-flow module (200 mL working volume) coupled with a PS ultrafiltration membrane. A centrifugal pump operating at 1 L/min was used to maintain the cross-flow of liquid in the vessel. As the outflow of the liquid sample from the reactor was pressure driven, the experimental system was operated at different values of transmembrane pressure ranging from 2 to 30 psi, and corresponding flux across the membrane was noted.

### Membrane studies

All experiments in the reactor were performed at room temperature (~28°C). For determining the physical adsorption of the dye (in the effluent) onto the membrane surface, the reactor was operated in the absence of laccase, and the absorbance of permeate was scanned over 450 nm to 650 nm. The percentage removal of the color in the effluent was calculated by measuring the area under the absorbance curve of the permeate samples collected at regular intervals during adsorption studies.

### Treatment of the effluent in EMR-batch and continuous studies

A bioreactor tank with 300 mL volume (200 mL working volume) and a permeate tank of 250 mL volume were used in the study. The O.D. of the combined effluent (Eff 5) used in the batch studies was 0.28 (between 510 and 550 nm) and corresponded to a dye concentration of ~90 ppm. An initial study was performed in which the effluent (without any enzyme addition) was allowed to come into contact with the membrane, and the filtrate was collected to determine its O.D. in the visible range. Once a constant O.D. was achieved in the permeate (~20 h, indicating no more adsorption of the dye on the membrane), laccase was added at 500 U/L working volume of the effluent. The experiments were run at different hydraulic retention times (HRTs) of 1.5 h, 3 h, 4 h, and 6 h. The pH of the effluent was adjusted to 4.0, and the reactor was operated at ambient temperatures (27–29°C). Permeate emerging out of the reactor was monitored at regular intervals for color removal, residual laccase activity, and toxicity by measuring the O.D., laccase activity, and colony count by the Ames test (as described under analytical methods). Average color removal at a specific HRT was calculated by taking an average of the % decolorization in samples tested at regular intervals. No additional laccase was added during the reactor operation. All experiments were carried out in triplicate, and the data given are an average (along with the standard deviation) of the reactor runs.

For continuous studies, an uninterrupted supply of the feed solution (Eff 6 or the test effluent) was maintained in the reactor with a peristaltic pump. The initial O. D. of the feed solution was 2.8 (at 610 nm), corresponding to a dye concentration of ~90 ppm. The pH of the effluent was adjusted to 4.0. The reactor was allowed to run for a period (~20 h) until the O.D. of the permeate (in the visible range) was constant. Laccase was added to an initial activity of 120–140 U/L of the test effluent and the reactor operated at an HRT of 6 h (based on the previously conducted optimization studies from the batch mode of reactor operation). Since there was a decrease in laccase activity as a function of time and it was proposed to be maintained between 120 and 140 U/L, two modes of addition were tested. In the first case, laccase was added after the initial activity decreased to ~10 U/L in the reactor, and in the other when the activity decreased to ~50 U/L in the reactor. Reactor samples were tested for laccase activity, while the permeate samples were tested at regular intervals for a reduction in dye concentration (measured as % decolorization) and toxicity. Based on the results obtained, the second regime of laccase addition was implemented, and the reactor operated at an HRT of 3 h and 6 h after the permeate O.D. was constant (~20 h). The systems were operated for an additional ~80 h after laccase addition. During the enzymatic treatment, the reactor samples were tested at regular intervals for laccase activity, and the permeate samples were tested for a reduction in color (or residual dye concentration) and toxicity (by the Ames test). Fouling of the membrane was determined by monitoring transmembrane pressure (TMP) vs. flux data before and after each run.

### Determination of the mutagenicity of the untreated and treated effluents

The mutagenicity of the treated effluent was assessed through the Ames test (Mortelmans and Zeiger, 2000). His^+^ revertants of *Salmonella typhimurium* strain TA 98 were observed after the incubation of the cells overnight in the plates containing different effluent samples. The samples tested included combined effluent before and after treatment with LCC1-62. In the control sample, the cells were plated on the medium without any additional liquid, and the number of colonies was counted after overnight incubation at 37°C. These represented spontaneous revertants. Colonies growing on the plates, containing the untreated effluent, were counted and represented the mutated cells. This number, adjusted with the spontaneous revertants, was taken as 100% mutagenicity of the sample. Colonies counted on the plates, containing the enzymatically treated effluent, represented the test samples. The average of the colonies (grown in triplicate) was used to calculate the reduction in mutagenicity. Percent inhibition was calculated by dividing the number of colonies obtained after enzyme treatment by the number obtained in the untreated sample × 100. The data were normalized with the number of spontaneous revertants.

### Analytical methods

The estimation of total protein in the crude culture filtrate and in the samples containing partially purified laccase was carried out according to Bradford (1976) using the Bio-Rad assay kit (bovine serum albumin was used as the standard). Laccase was measured using ABTS as a substrate according to Eggert et al. (1996). One unit of enzyme activity was defined as an amount that catalyzed the conversion of 1 μmol of ABTS radical cation (ABTS^*^+) in 1 min. The molar extinction coefficient of ABTS was taken as 36,000 cm^−1^ M^−1^.

### Statistical analysis

Statistical analysis of the data obtained on the enzymatic treatment of different effluents and the mutagenicity was carried out by ANOVA using GraphPad Prism version-5 (GraphPad Software, San Diego, CA, USA). A *p*-value of ≤ 0.05 (level 5%) was adopted as a significance criterion. Statistical analysis of the data obtained on the variance of the dye concentration under different HRTs (1.5, 3, 4, and 6 h) was performed using the F-test.

## Results

### Production of LCC1-62 and partial purification of laccase

Production of extracellular laccase was carried out by cultivating the recombinant *P. pastoris* strain in BMMG followed by cultivation in the BMMY medium as described (Kenzom et al., 2014). Methanol was added every 24 h to 1% (v/v) to maintain the induction of the mutated *Lcc1* gene, coding for the laccase variant LCC1-62, under the control of the alcohol oxidase 1 promoter. The extracellularly produced laccase was purified by the ultrafiltration of the cell-free broth without any treatment. The specific activity of the WTLCC1 and LCC1-62 in the crude culture filtrate was 8.13 U/mg extracellular protein and 8.11 U/mg extracellular protein, respectively. After ultrafiltration, the specific activity was 13.74 U/mg protein with a yield of ~60% for the WTLCC1. For LCC1-62, the specific activity was increased to 11.17 U/mg protein with a yield of 63%. The enzyme was kept at −20°C for long-term storage where the activity was retained by over 90% over a period of 6 months. Aliquots were removed whenever required for running the experiments.

### Evaluation of laccase on different effluents

**Figure 2:**
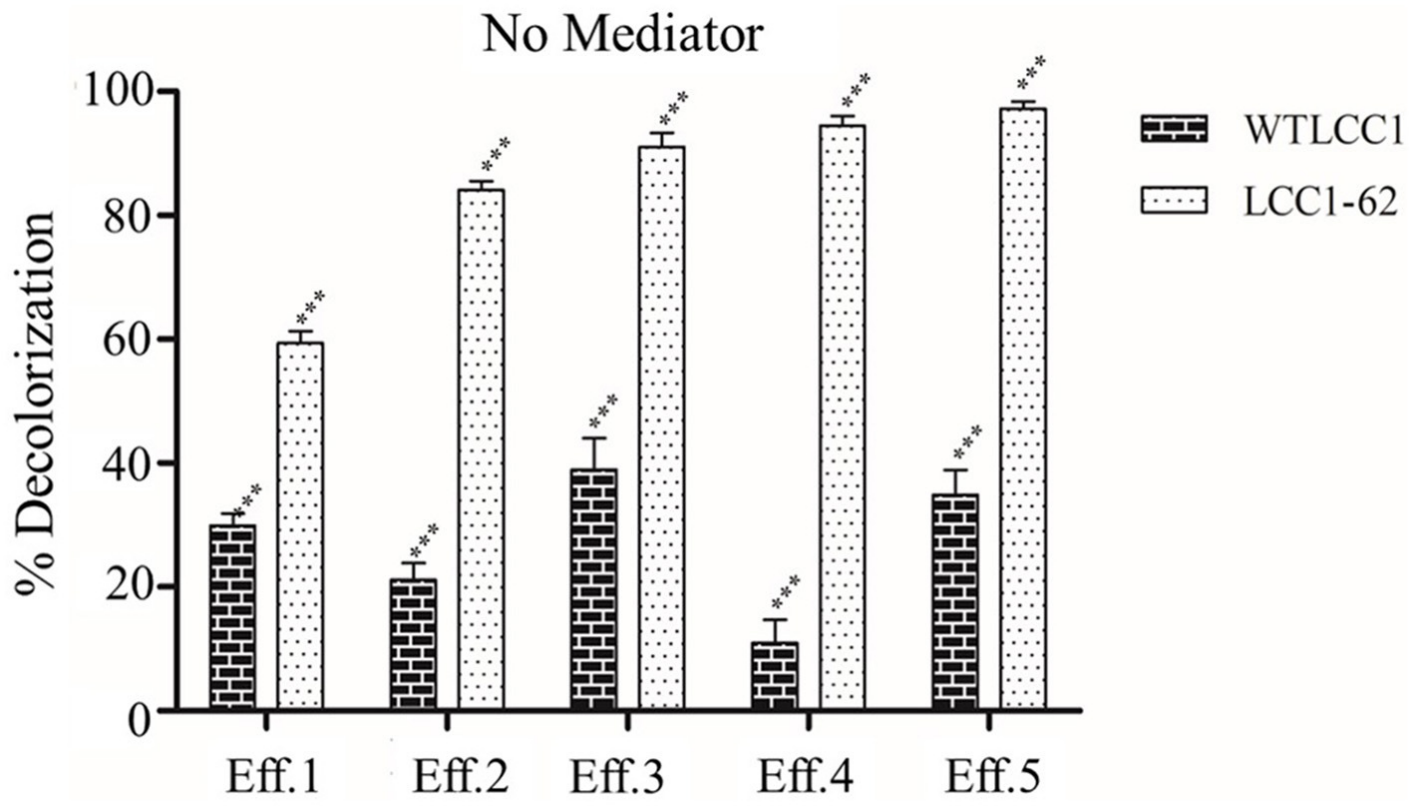
Decolorization data obtained by the treatment of five effluents. Percent decolorization of Effluents Eff 1–Eff 5 when treated with either WTLCC1 or the hyper-active variant LCC1-62 in the absence of any mediator. Statistical analysis of the data was carried out by ANOVA using GraphPad Prism software. A *p*-value of ≤ 0.05 (level 5%) was adopted as the significance criterion and the calculated *P*-value for the source of variation through “Interaction”, “Column Factor”, and “Row Factor” was <0.0001 (*P* < 0.0001), indicating significance (***) of the results.

The efficacy of decolorization of the partially purified LCC1-62 was first evaluated on different effluents, and the data are shown in Figure 2. As seen, laccase was effective on all the five effluents, and between 60 and 95% decolorization was achieved with LCC1-62 including its effectiveness on Eff 1, the dyeing bath effluent. The WTLCC1, on the other hand, was less effective (<40% decolorization) on the effluents. No mediators were added to the samples. As shown in Supplementary Figure S3, the engineered LCC1-62 effectively (>95%) decolorized Eff 5, which contained predominantly Disperse Red 73 in approximately 12 h. Eff 5 was used for studies conducted in batch mode while the test effluent was used during the continuous study.

### Characterization of membrane fouling

An important factor that ensures stable tangential flow filtration (TFF) is the designing of control permeate flux to certain reasonable levels that may result in reduced fouling and polarization. A preliminary study was performed in the membrane reactor to define the hydro-dynamic conditions in which the colloidal particle deposition was checked on the membrane surface. An initial control run was performed with clean water, and the flux across the membrane was noted at different TMPs ranging from 2 to 30 psi. Flux was also measured in a solution containing 500 U/L of partly purified laccase or Eff 5. As shown in Supplementary Figure S4, a slight reduction (~5%) in flux was noted for the enzyme-containing solution, compared to the Eff 5 sample, till 20 psi TMP. At higher TMP, the flux was reduced by ~15%. The flux was again measured after running the reactor, containing only the Eff 5, for 6 and 24 h. No significant difference was observed in the flux, indicating membrane fouling to be negligible by the untreated Eff 5. Longer operation of the reactor for 2–3 days did not indicate any membrane fouling. However, the flux was decreased only after long operations (>15 days), and the difference was more significant at TMP above 12 psi. Thus, it was concluded that the colloidal matter in the effluent did not affect flux across the membrane, and thus membrane fouling was minimum.

### Treatment of the combined effluent-control study on the membrane

For determining the physical adsorption of the dye onto the membrane surface, a controlled study was performed by running the reactor in the absence of laccase and measuring the absorbance of the permeate sample at regular intervals of time. As shown in Supplementary Figure S5, no dye was observed initially in the permeate sample. The samples collected at later time points showed the presence of the dye (absorption in the region of 490 to 560 nm) in the permeate which overlapped with the absorption spectrum of the combined effluent. The O.D. of the permeate samples increased with the passage of time, indicating that the dye from the effluent sample (i) adsorbed on the membrane surface during the initial hours of the run, (ii) once saturation was reached, it crossed the membrane and appeared in the permeate, and (iii) the system attained equilibrium within ~20 h. The percentage increase in color (Supplementary Figure S5) in the permeate samples was calculated by taking the area under the absorbance values shown from 510 to 530 nm. The control study was concluded with the observation that after an initial period, the dye adsorption onto the membrane surface ceased, and the system operated under stable conditions.

### Treatment of the combined effluent in EMR-batch studies

**Figure 3:**
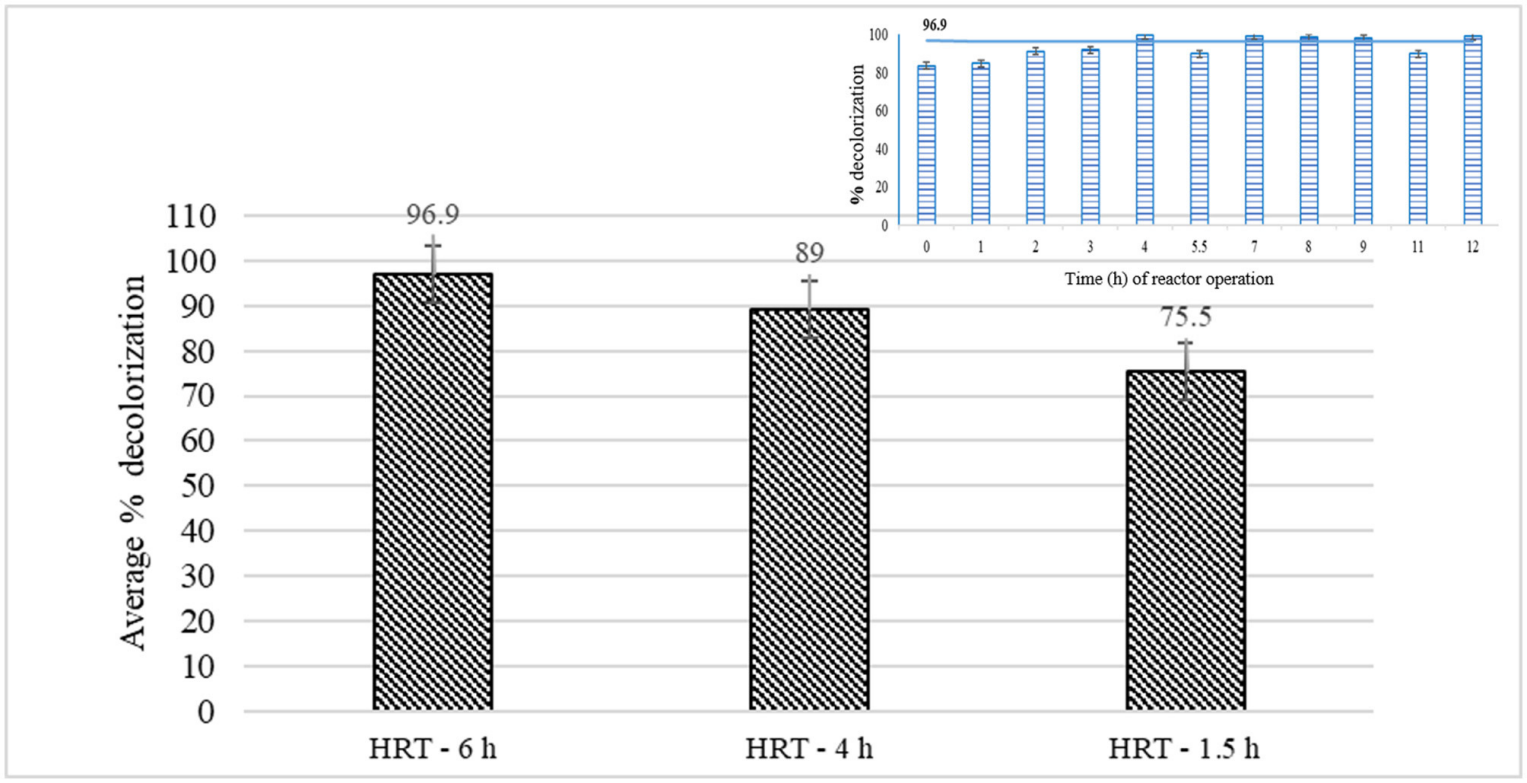
Percent decolorization of the combined effluent in batch reactor studies at different hydraulic retention times. The inset shows how the average values were calculated after removing the samples at regular intervals followed by monitoring decolorization during a particular run.

The treatment efficiency of the laccase variant, LCC1-62, was studied in a ray-flow membrane reactor with 200 mL working volume in batch mode at different HRTs. The absorbance of the permeate, emanating without any enzyme treatment, was observed during the initial hours and found to increase up to a period of 20 h after which it was constant, indicating that a fraction of the dye molecules adsorbed on the membrane. The absorption spectrum of the permeate after attaining equilibrium is shown (Supplementary Figure S6, panel 1). Treatment with laccase reduced absorption at all wavelengths indicating the action of laccase on the dye(s). The actual color, before and after treatment with laccase, is shown in Supplementary Figure S6 (panels 2 and 3). The removal of color was observed to increase with an increase in the HRT from 1.5 h to 6 h. Percent decolorization was the same at HRT of 3 (data not shown) and 4 h with the highest average decolorization of 97% (residual dye concentration of ~3 ppm) at HRT of 6 h (Figure 3). The inset in the figure shows the computation of the average removal of the dye during the reactor operation for which the samples were removed after every hour, and O.D. was measured between 410 and 710 nm. The decrease in the area under the curve was compared with that obtained from the untreated sample to calculate percent decolorization at that time point. Importantly, the PS membrane was effective in retaining the enzyme in the reactor, and no enzyme activity was observed in the permeate samples. The enzyme stability in the reactor was studied over each run at different HRTs with samples being removed from the reactor vessel. An increasing trend in enzyme activity per ml of bioreactor sample was seen as a function of time during the first few hours of reactor operation, due to the recirculation of the filtrate into the reactor. Longer operation times led to a loss of laccase activity. Thus, periodic addition of laccase was initiated in continuous reactor runs.

### Treatment of the combined effluent in EMR-continuous reactor studies

**Figure 4:**
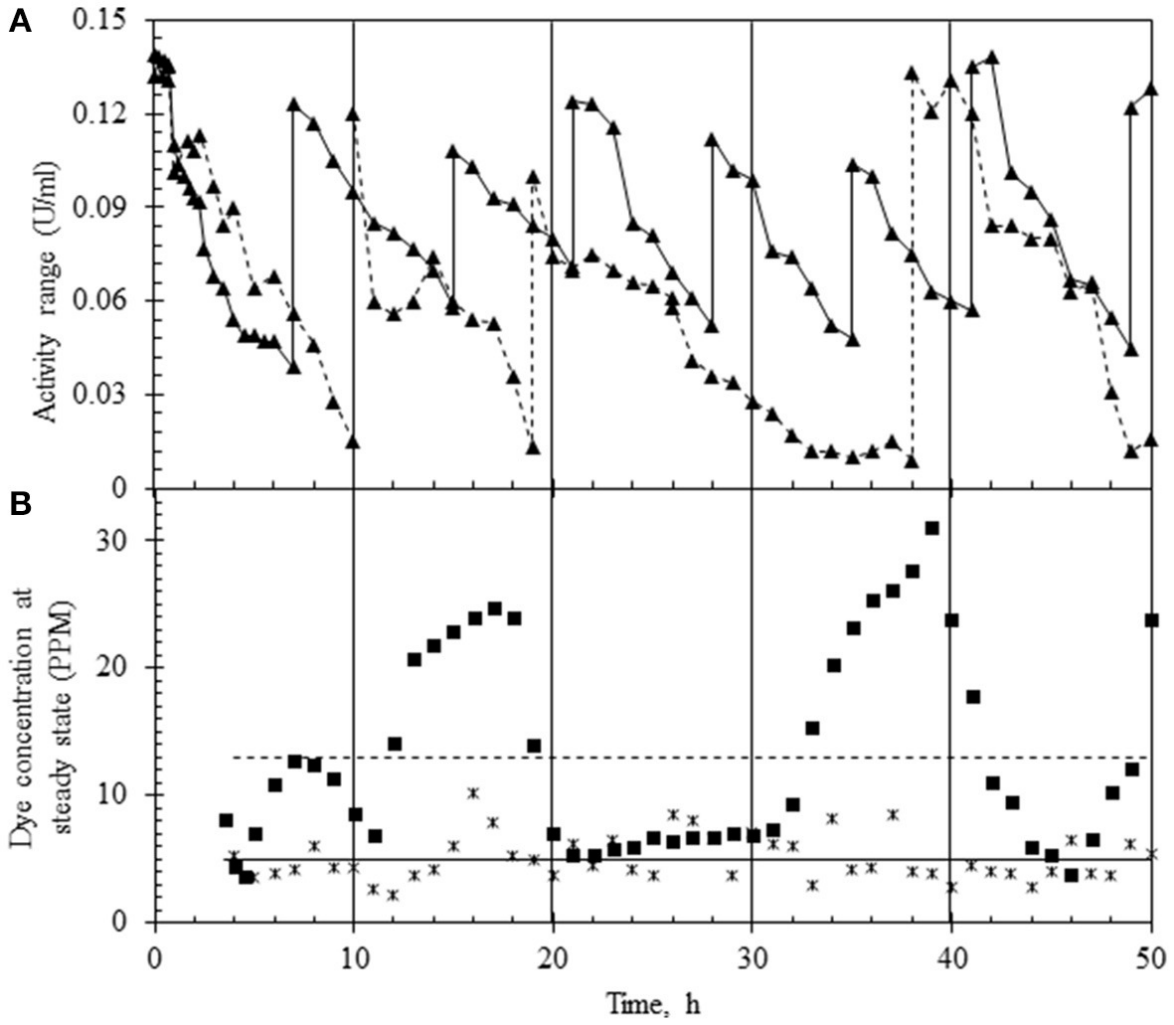
Laccase activity profile and variance of dye concentration in two different modes of continuous reactor operation (at HRT of 6 h). **(A)** Laccase activity profile based on the replenishment of enzyme in the reactor which was (i) between 140 and 50 U/L (▴) or (ii) 140 and 10 U/L (–▴-). **(B)** Concentration of the dye in the permeate based on the enzyme replenishment regime which was (i) between 140 and 50 U/L (*) or (ii) 140 and 10 U/L (■). The mean concentration of the dye under the two regimes is shown as a line passing through the data points.

A preliminary study was conducted to pick the right range of laccase activity over which the reactor must run to obtain maximum % decolorization. For this, the reactor was operated at an HRT of 6 h with the upper limit of laccase adjusted between 120 and 140 U/L. The lower limit was decided based on the residual dye concentration, and the variance in the dye concentration was obtained by following two different strategies of laccase addition. In the first case, laccase was replenished when activity in the reactor decreased by 40% (to ~50 U/L) (Figure 4A), while in the second case, laccase was injected when the activity decreased by ~90% of the initial value (to ~10 U/L) as shown (Figure 4A). In both modes of operation, fluctuation in the dye concentration was noted in the permeate. As seen in Figure 4B, the variance (σ^2^) in the dye concentration for laccase activity ranging from ~120–50 U/L (enzyme replenishment after 40% fall of initial enzyme activity) was 2.77 with an average of 5 ppm residual dye concentration, corresponding to 95% decolorization. In the second case, a higher variance (σ^2^) in the dye concentration (62.08), with an average residual dye concentration of 13 ppm (Figure 4B), corresponding to 86% dye decolorization was observed. The data suggested the lower limit of laccase be maintained at ~50 U/L to get nearly complete dye removal.

**Figure 5:**
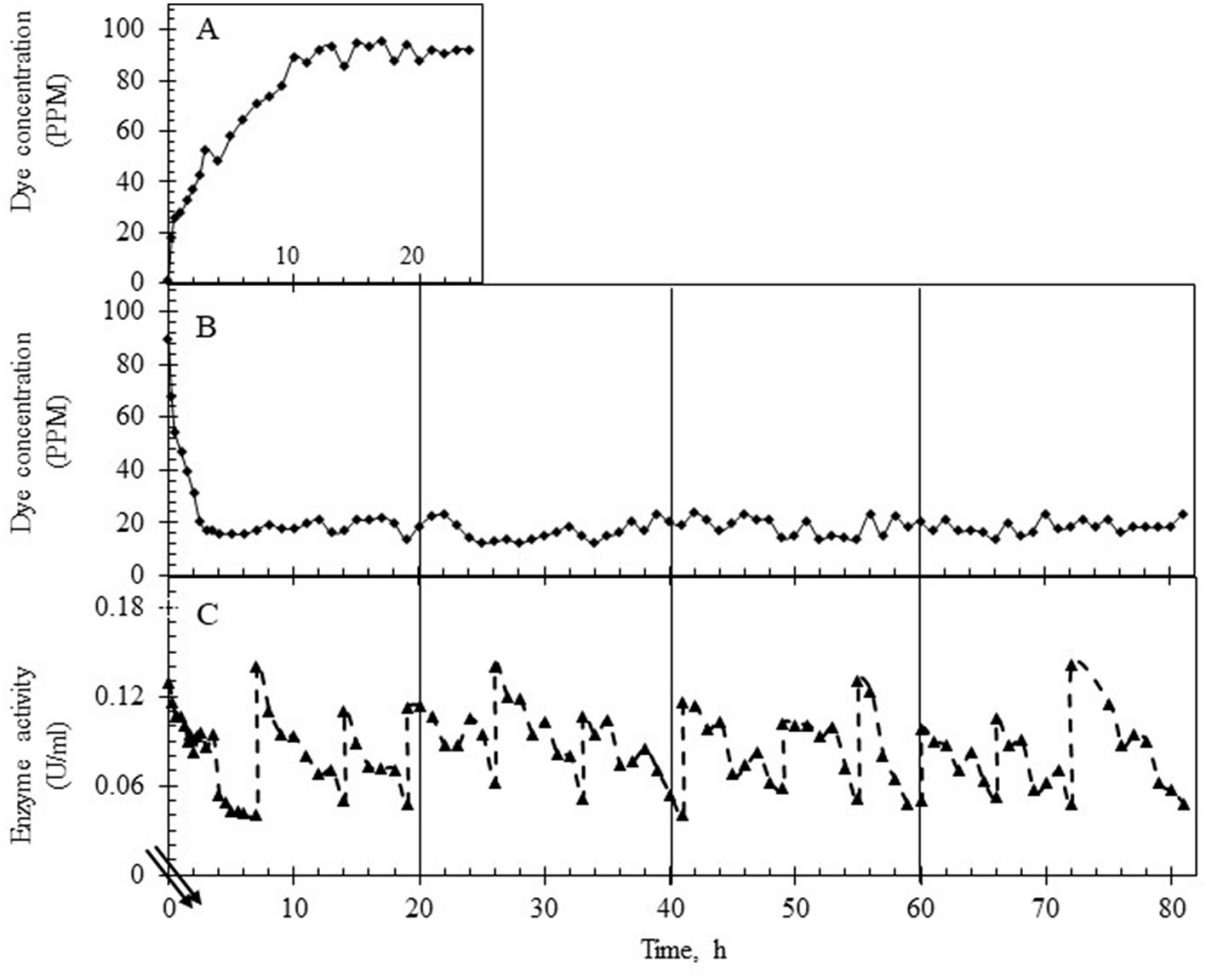
Dye and laccase activity profile during continuous operation of the reactor operating at an HRT of 3 h. **(A)** Dye concentration (in ppm) in the permeate till equilibrium was attained, ~20 h (control study). **(B)** Dye concentration (in ppm) in the permeate after laccase addition. **(C)** Enzyme activity profile in the reactor during the reactor operation with intermittent laccase addition to restore the activity to ~140 U/L. The double lines in **(C)** show the time after the initial 20 h.

**Figure 6:**
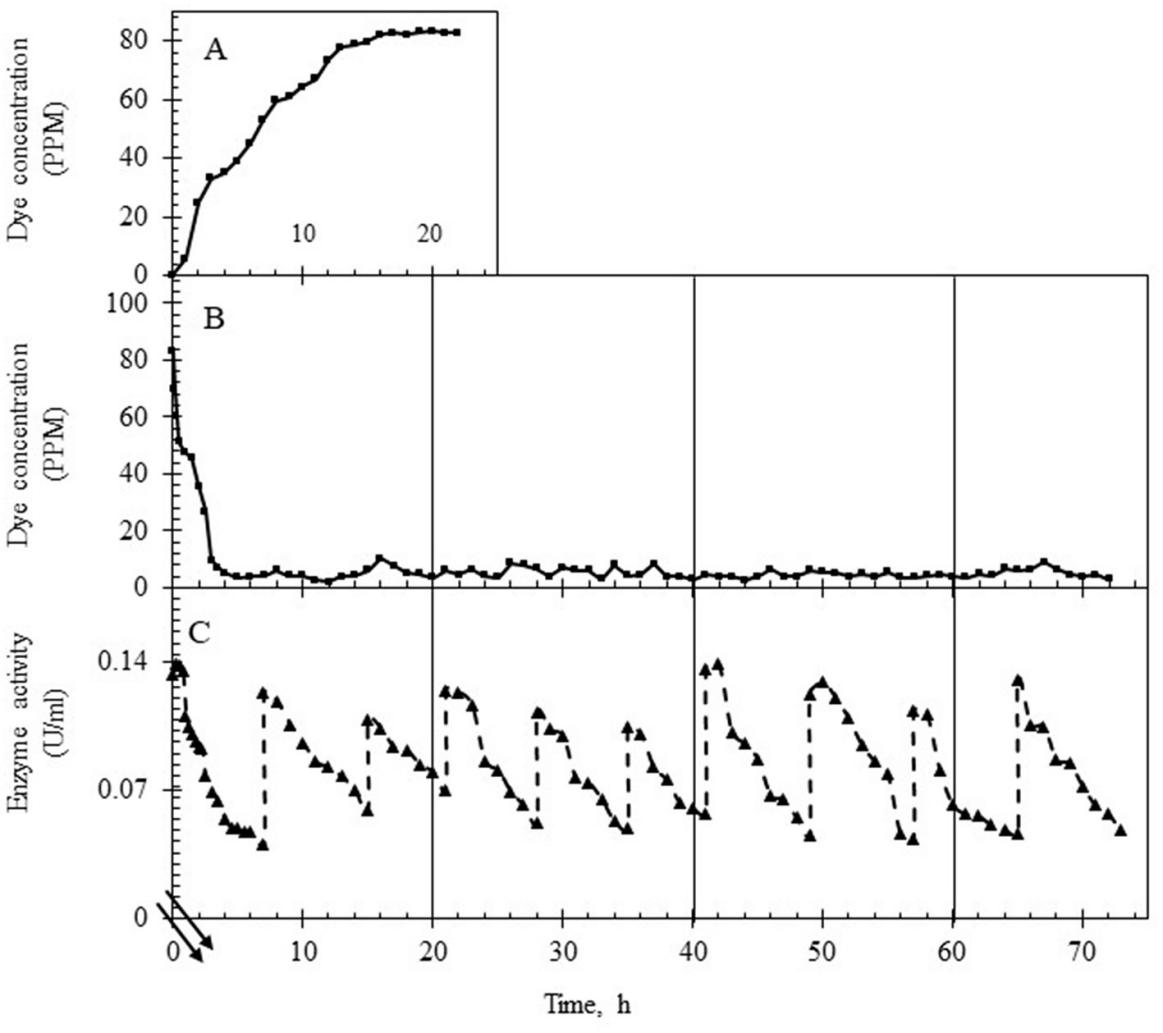
Dye and laccase activity profile during continuous operation of the reactor operating at an HRT of 6 h. **(A)** Dye concentration (in ppm) in the permeate till equilibrium was attained, 20 h (control study). **(B)** Dye concentration (in ppm) in the permeate after laccase addition. **(C)** Enzyme activity profile in the reactor during the reactor operation with intermittent laccase addition to restore the activity to ~140 U/L. The double lines in **(C)** show the time after the initial 20 h.

**Figure 7:**
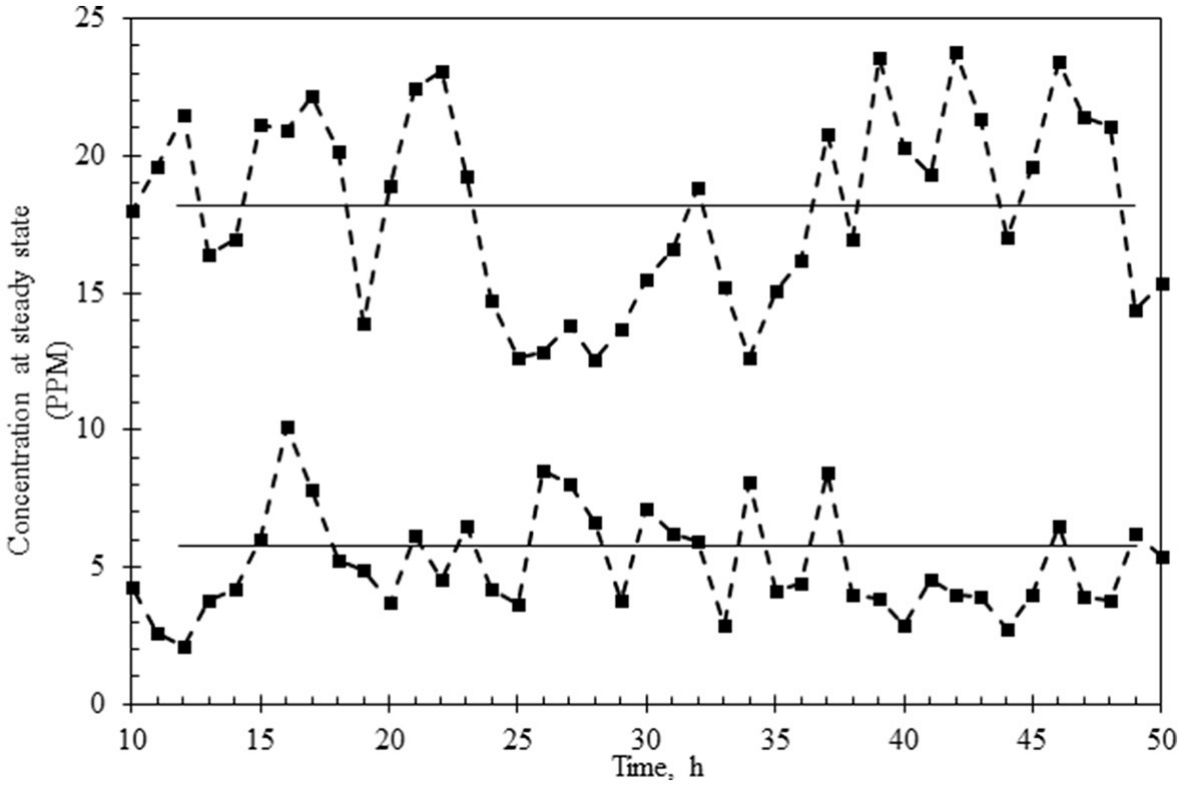
Variance of the dye concentration under a pseudo-steady-state of reactor operation at HRT of 3 h **(upper graph)** or 6 h **(lower graph)**. The mean concentration of the dye is shown as a straight line in the data points.

The continuous reactor was operated with the laccase supplementation strategy (replenishment to maintain 50 U/L laccase) at HRTs of 3 h and 6 h for over 3 days to assess the effect of retention time on (i) % decolorization, (ii) variance in the dye concentration, (iii) state of TDS in the reactor and in the permeate, (iv) membrane fouling, and (v) detoxification. As shown in Figures 5A, 6A, the system was first allowed to reach equilibrium (~20 h) till the dye concentration in the reactor was steady between 85 and 95 ppm. The addition of laccase (to 120–140 U/L) resulted in an increase in % decolorization from 86 to 94% as HRT increased from 3 to 6 h with a residual concentration of ~18 ppm and ~5 ppm, respectively (Figures 5B, 6B). The enzyme activity in the retentate was monitored continuously and maintained between 120 and 140 U/L as seen in Figures 5C, 6C. Samples collected at different time points (3–80 h post stabilization of the dye concentration in the reactor) showed an almost overlapping spectrum indicating nearly uniform conditions (pseudo-steady-state) in the continuous experiment. The observed pseudo-steady-state was observed because of the decrease in enzyme activity in the reactor with time which had a strong effect on the quality of the treated wastewater. This was measured in terms of the variance in color in the permeate samples (treated wastewater), collected during the reactor run. The variance in the dye concentration when the reactor was operated at 3 h and 6 h for 3 h was 9.89 and 2.80 respectively. This indicated that the % decolorization and the variance were more sensitive to lower HRT (or high feed rate). A similar trend of increase in variance was observed when the enzyme activity range was increased for the 6 h HRT (Figure 4), indicating that % decolorization was also more sensitive to enzyme activity variation than the HRT. The details of the actual dye concentration along with the mean dye concentration during the reactor operation are shown in Figure 7. Variance in the dye concentration in the permeate, emanating after the laccase treatment, is an important factor and it was observed that the variance in the dye concentration in the permeate was ~three times higher when the reactor was operated at an HRT of 3 h compared to 6 h.

**Figure 8:**
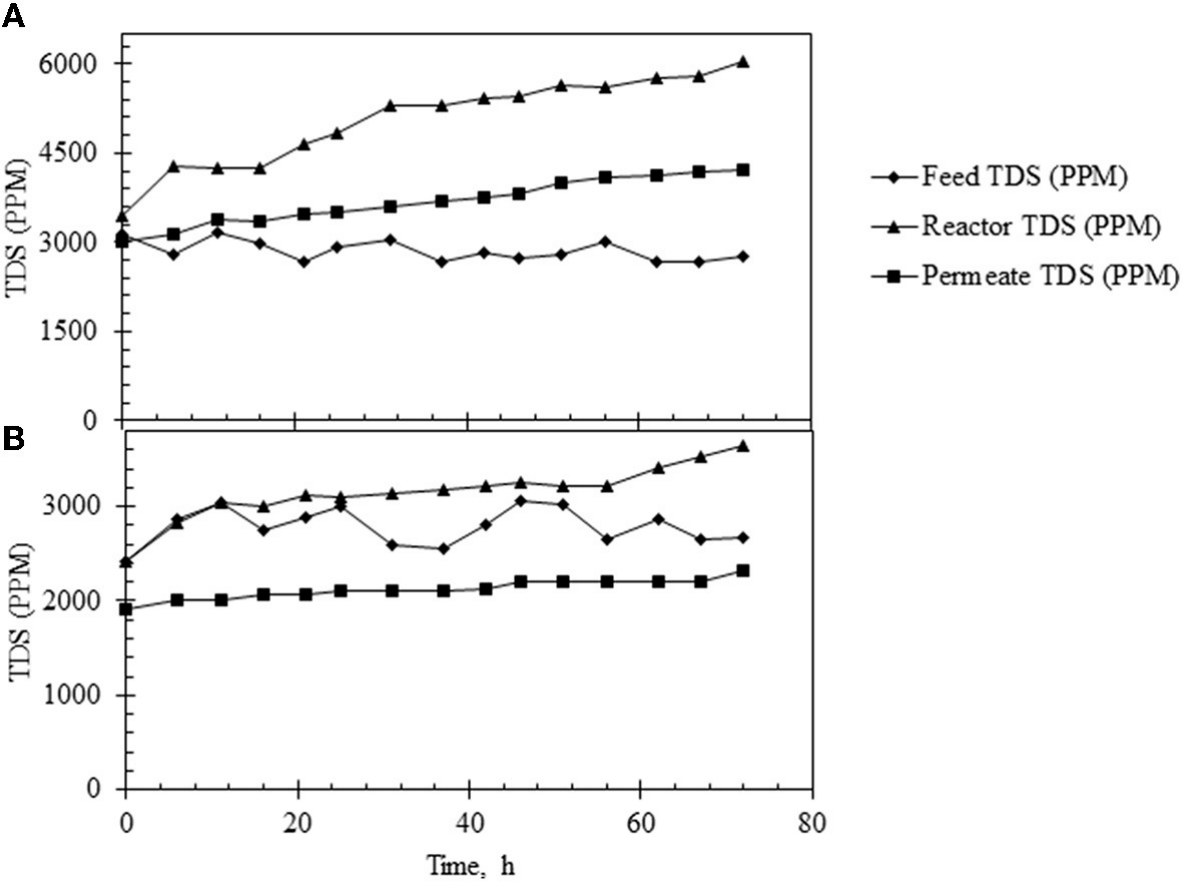
TDS profile under different operating conditions in the continuous reactor. **(A)** TDS profile in the feed (♦), reactor (▴), and permeate (■) under continuous reactor operations at HRT of 3 h. **(B)** TDS profile in the feed (♦), reactor (▴), and permeate (■) in continuous reactor operating at HRT of 6 h.

It was observed that the TDS in the feed was constant (at ~3,000 ppm) but it increased steadily in the reactor with time when the HRT was 3 h. This consequently led to an increase in the TDS of the permeate. When the reactor was operated at an HRT of 6 h, constant TDS was maintained in the permeate at approximately 2,500 ppm (Figures 8A, B). The membrane performance was also evaluated, and the results are shown in Supplementary Figure S7. As shown, the membrane performance was poorer when the reactor was run at an HRT of 3 h.

### Toxicity analysis of the effluent

The untreated and the treated samples were tested in duplicates and the number of colonies counted. Milli-Q water was taken as control, and colonies developed in control plates were the spontaneous revertants (an average of 58 colonies). The average number of colonies in the untreated samples was ~5,000. Normalized to the spontaneous revertants (58), 4,942 colonies represented 100% mutagenicity. After enzymatic treatment, the average number of colonies, normalized to spontaneous revertants, at 6 h HRT, was 171 and 122 in batch and continuous mode of reactor runs, respectively (Table 1). This indicated a reduction in the mutagenicity of the treated samples by 96.5 and 97.5%, respectively. Thus, the results indicated effective treatment of the effluent with LCC1-62.

**Table 1:** Mutagenicity of the untreated and the treated combined effluent as tested by the Ames test.

**Sample**	**No. of colonies**				
	**Plate 1**	**Plate 2**	**Average**	**Normalized**^#^ **to control**	**% inhibition**
Control (Spontaneous revertants)	81	35	58	-	-
Combined effluent (untreated)	~5,000	~5,000	5,000	4,942^*^	100
Permeate, HRT 1.5 h	397	371	384	326	6.6
Permeate, HRT 4.0 h	344	328	336	278	5.6
Permeate, HRT 6.0 h	250	208	229	171	3.5
Permeate, HRT 6.0 h (continuous operation)	204	157	~180	122	2.5

The permeate samples are from either the batch operation at the indicated HRT or from the continuous operation. ^*^This is taken as a 100% inhibition value. The inhibition of the treated samples is compared to this value for determining % inhibition. ^#^The number of spontaneous revertants obtained in the control sample is subtracted from the average of the sample colonies.

## Discussion

Effluents generated from the textile industry pose a considerable threat to the environment as the dyes are toxic, recalcitrant, resistant to decolorization, and have been reported to be mutagenic and carcinogenic (Patil et al., 2022). For the treatment of textile wastewater, three types of process technologies, largely based on physical, chemical, and advanced oxidation methods (Imran et al., 2015) have been developed and these are (i) separation and concentration, (ii) decomposition and degradation, and (iii) exchange of materials (Bechtold et al., 2004). Since these led to the generation of sludge, biological methods have gained attention (Ali, 2010; Sosa-Martínez et al., 2020; Coria-Oriundo et al., 2021; Al-Tohamy et al., 2022). In the present study, the previously reported (Kenzom et al., 2014) catalytically superior laccase variant LCC1-62 (with higher K_cat_) of LCC1, one of the many isoforms of laccase (Ahlawat and Mishra, 2022), was used. The engineered LCC1-62 was produced in the recombinant *P. pastoris*, and the partially purified laccase was used for the treatment of real textile effluent in an EMR. The membrane-based technologies have shown superior performance in the treatment of domestic wastewater and a wide range of effluents from several industries (Jegatheesan et al., 2016; Al-Asheh et al., 2021; Yang et al., 2021). For instance, ~80% removal of Remazol Brilliant Blue R was achieved in an EMR using laccase from *Trametes versicolor* with the aid of syringaldehyde (Mendoza et al., 2011). In another study, a highly thermo-, metal-, surfactant-, and organic solvent-tolerant laccase from *Bacillus* sp. MSK-01 was immobilized in Cu-alginate beads along with ABTS and used in a continuous-flow packed-bed bioreactor for continuous treatment of effluent. Approximately 66% reduction in color was achieved with the concomitant breakdown of dyes into degradation products (Thanh et al., 2012). Nguyen et al. (2014) reported a 60% degradation of carbamazepine, sulfamethoxazole, and atrazine using laccase from *Aspergillus oryzae* in the presence of syringaldehyde during the continuous mode of operation in an EMR. In another study (Yin et al., 2019), error-prone PCR was used for generating an alkali-tolerant laccase which decolorized indigo carmine at higher pH (7.0–7.5) compared to a commercially available laccase from *Trametes villosa*. An engineered laccase from *Bacillus licheniformis* was shown to decolorize acid violet by 78% compared to 40% by the wild-type form (Bu et al., 2020). Thus, engineered laccase from fungi and bacteria has shown promise for application in textile industries. In all these studies, the feasibility of using EMR was demonstrated with laccase in the presence of mediators. The main drawback of using mediators is that they add to the cost of operation and increase the toxicity of the effluent. Thus, mediator-free treatment is desirable.

In this study, we report the use of the engineered LCC1-62 variant for the treatment of the combined effluent containing diverse azo dyes at ~90 ppm level without the need for any mediator. Batch operations allowed us to determine the laccase dosage that could be used in the continuous reactor which was ~120–140 U/L and the HRT for maximum decolorization. One of the important observations was that the dyes adsorbed onto the membrane in the initial phase of the reactor operation and this continued for ~20 h. This can be avoided by the modification of the membrane. Second, while the enzyme did not appear in the permeate, there was a loss of laccase activity in the reactor, which had to be replenished when the levels decreased to ~40% of the original activity. This was attributed to shear stress or decay in enzyme activity in the presence of salts or other auxiliaries in the effluent (Arregui et al., 2019). Allowing the laccase activity to decrease to 90% of the original dosage resulted in less % decolorization and greater variance in the dye concentration as opposed to supplementation when the residual activity decreased to 40% of the original dosage. The performance of the reactor, in terms of dye decolorization, was also sensitive to HRT as lower HRT (3 h) resulted in lesser % decolorization and greater variance in dye concentration than a higher HRT of 6 h. Higher HRT favored effective laccase action on the dyes in the effluent, and the polymerization activity of laccase was found to be reduced under these conditions. This also resulted in less membrane fouling during the period of operation of the reactor. The third novelty of the study was the use of free laccase. The immobilization methods often used to increase laccase stability affect the catalytic activity on account of restricted laccase–substrate contact leading to slowing of the decolorization process (Gioia et al., 2018; Zdarta et al., 2021). While significant improvements were shown for catalytic activity and dye affinity when laccase was cross-linked by glutaraldehyde to silica-coated magnetic multiwalled carbon nanotubes (Fe_3_O_4_-MWCNT@SiO_2_) and immobilized, there was a loss of enzyme after a few cycles of re-usage (Habimana et al., 2021).

A combination of chemical/physicochemical/advanced oxidation and biological methods has also been suggested for the treatment of textile effluents. In a recent study (Aydin et al., 2021), a combination of biological (cell-based) followed by photo-electrocatalysis was used for desalination, disinfection, and toxicity removal. While the advantage of such a method is that it allows the reuse of water, the biological systems eventually cease to grow in the effluent and result in the choking of the membrane. Furthermore, the reduction in toxicity is not of the desired level. In another study (Yang et al., 2021), a laboratory-scale pilot plant of a moving bed biofilm reactor was coupled with a membrane bioreactor and evaluated for the treatment of textile wastewater. COD removal by 93% and decolorization by 85% were achieved. Additionally, 99% of TSS was removed due to filtration but resulted in the accumulation of sludge. More recently, a metal-organic framework material, namely, zeolitic imidazolate framework-90 (ZIF-90), was used for the encapsulation of laccase, and combining this with bacterial cellulose and carbon nanotubes led to the formation of a novel membrane with catalytic function which degraded phenolic pollutants (Li et al., 2021). Anaerobic reactors have also been successfully used for the removal of textile dyes and reported to result in a COD reduction of 95% and dye removal by 97%. The main disadvantage of such processes is the accumulation of extracellular polymeric substances in the effluent (Berkessa et al., 2020).

Some major points of concern in this study, observed in the long-term operation of the EMR, were (i) membrane fouling, (ii) laccase replenishment, and (iii) an increase in TDS in the reactor. Several solutions have been suggested to overcome membrane fouling such as the pre-treatment of the membrane by (i) the addition of powdered activated carbon which can adsorb solids and colloids (Park et al., 1999), (ii) the addition of powdered activated charcoal and granular activated carbon (Hu and Stuckey, 2007), (iii) the addition of coagulants, and/or (iv) the addition of alum and powdered activated charcoal (Thanh et al., 2012; Du et al., 2020). Alternatively, nanomembranes could be used to place the enzyme in the reactor so that their adsorption or entry into the pores of the membrane can be prevented. The second problem of retention of enzyme activity can be addressed by subjecting the recombinant LCC1-62 to a second round of mutagenesis followed by a selection of active variants in the presence of effluent. Cross-linking of laccase can also be carried out as many enzymes have shown increased stability toward high temperature, inorganic and organic contaminants, and biological denaturants in the aggregated form (Matijošyte et al., 2010; Ba et al., 2012). Treatment with low alum/other coagulants can be attempted to reduce the TDS in the incoming feed. The combination treatments have been reported to be particularly valuable in the treatment of several categories of effluents (Chhabra et al., 2015a,b; Deng et al., 2020).

## Conclusion

The effectiveness of a laccase hyper-catalytic variant LCC1-62 of the LCC1 isoform of *C. bulleri* was shown in removing color and toxicity from several effluents obtained from denim dyeing, cotton, and polyester dying mills. Evaluation of this variant on a combined effluent obtained from a mill using diverse azo dyes, in an enzyme membrane reactor, indicated more than 95% decolorization without the aid of any mediator. Loss of laccase activity (~40%) after initial hours of operation was compensated for by the injection of fresh laccase in a way such that the variance in the dye concentration was minimized and effective decolorization achieved. The reactor operated at several HRTs indicated performance to be better at an HRT of 6 h. The operation of the continuous reactor with enzyme replenishment led to successful operation with very little membrane fouling. The process was accompanied by more than 90% reduction in toxicity. The usage of laccase was lower than reported in earlier studies and is the first with a free-engineered laccase in an EMR. Given this data, two important things emerge. First, large-scale production of laccase at an economical price is required which seems feasible due to the availability of laccase from recombinant *P. pastoris*. Second, either very high titers of enzyme in a liquid medium or spray-dried preparations of active laccase are required. The latter has been prepared and tested in our laboratory and shows promising results.

## Data availability statement

The original contributions presented in the study are included in the article/Supplementary material, further inquiries can be directed to the corresponding author.

## Author contributions

MD: conceptualization, experimental execution, and writing—original draft. DI: experimental execution and data analysis. PS: supervision and data analysis. TS: supervision, data analysis, and resources. SM: conceptualization, data analysis, supervision, resources, writing—reviewing, and preparation of the final manuscript. All authors contributed to the article and approved the submitted version.
