# Supplementary material for: Detoxification and decolorization of complex textile effluent in an enzyme membrane reactor: batch and continuous studies
Source: Front Microbiol. 2023 Jul 7;14:1193875. doi: 10.3389/fmicb.2023.1193875 (PMC10361525; doi:10.3389/fmicb.2023.1193875)

Supplementary Table S1. Composition of the effluents

Characteristic Eff 1 Eff 2 Eff 3 Eff 4 Eff 5 Eff 6

pH 4.5 5.8 6.0 6.6 9.5 9.8

Conductivity (micromhos/cm) 5000 6400 8500 9000 n.a. n.a.

TDS (mg/L) 540 5500 6500 7700 ~3000 ~3000

COD (mg/L) 9000 620 650 750 1000 1250

TSS (mg/L) n.a. 55 85 120 90 120

Color (Pt/Co units) n.a. 76 800 1200 n.a. n.a.

Dye concentration (mg/L) 570 (IC) 80 (IC) 100 (IC) 97 (IC) ~90 (DR 73, ~90 (DB

DY 211) 173)

n.a.: not available, IC: indigo carmine, DR: disperse yellow, DY: disperse yellow, DB: disperse blue.

The source of the effluents is described in the text.

Supplementary Fig. S1. Structure of the dyes present in the combined effluent used in batch and continuous studies

**Disperse Yellow 211**


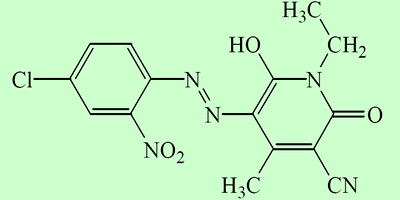


λ_max_:496 nm

**Disperse Red 73**


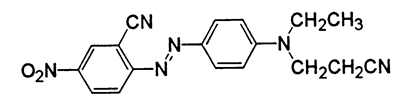


λ_max_:530 nm

**Disperse Blue 173**


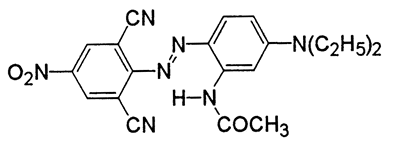


λ_max_:600 nm

Supplementary Fig. S2. The ray-flow membrane reactor used in the study. The conical flask (on the left) shows the combined effluent being fed in the membrane reactor while the flask on the right shows the decolorized permeate emerging after the enzyme treatment


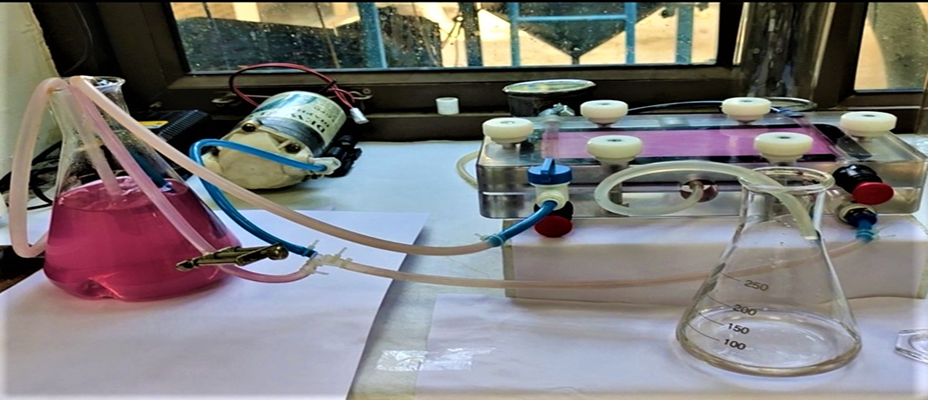


Supplementary Fig. S3. Decolorization of different effluents after treatment with the WTLCC1 or LCC1-62 (at 100 U/L reaction mixture). The time at which the maximum decolorization was achieved is shown above the arrows.


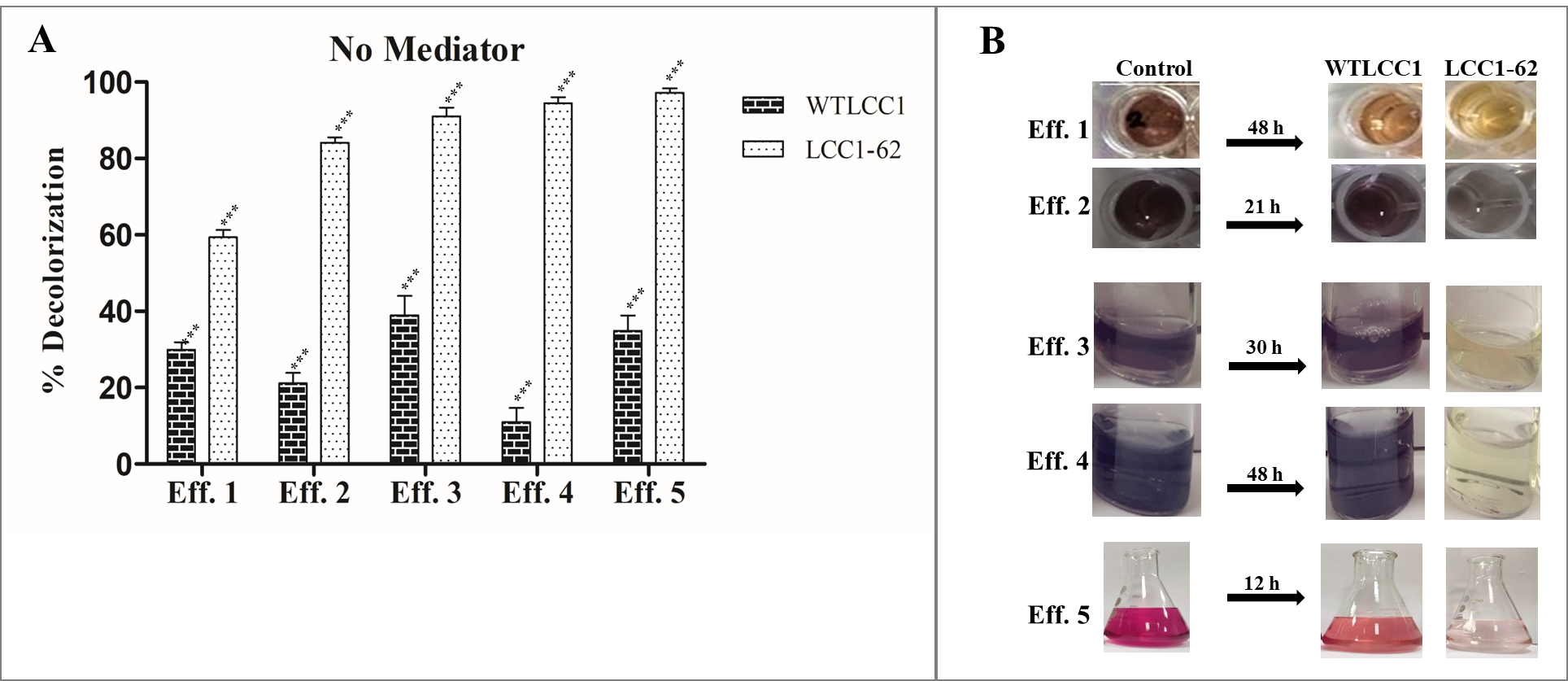


Supplementary Fig. S4. Fouling characterization of the PES membrane using combined effluent. The permeate flux was recorded as a function of TMP. The permeate function was also recorded in samples containing the enzyme solution (500 U/L). The duration of the reactor operation is shown in the parenthesis in the inset. Black solid squares show the relation between flux and TMP of clean water (control).


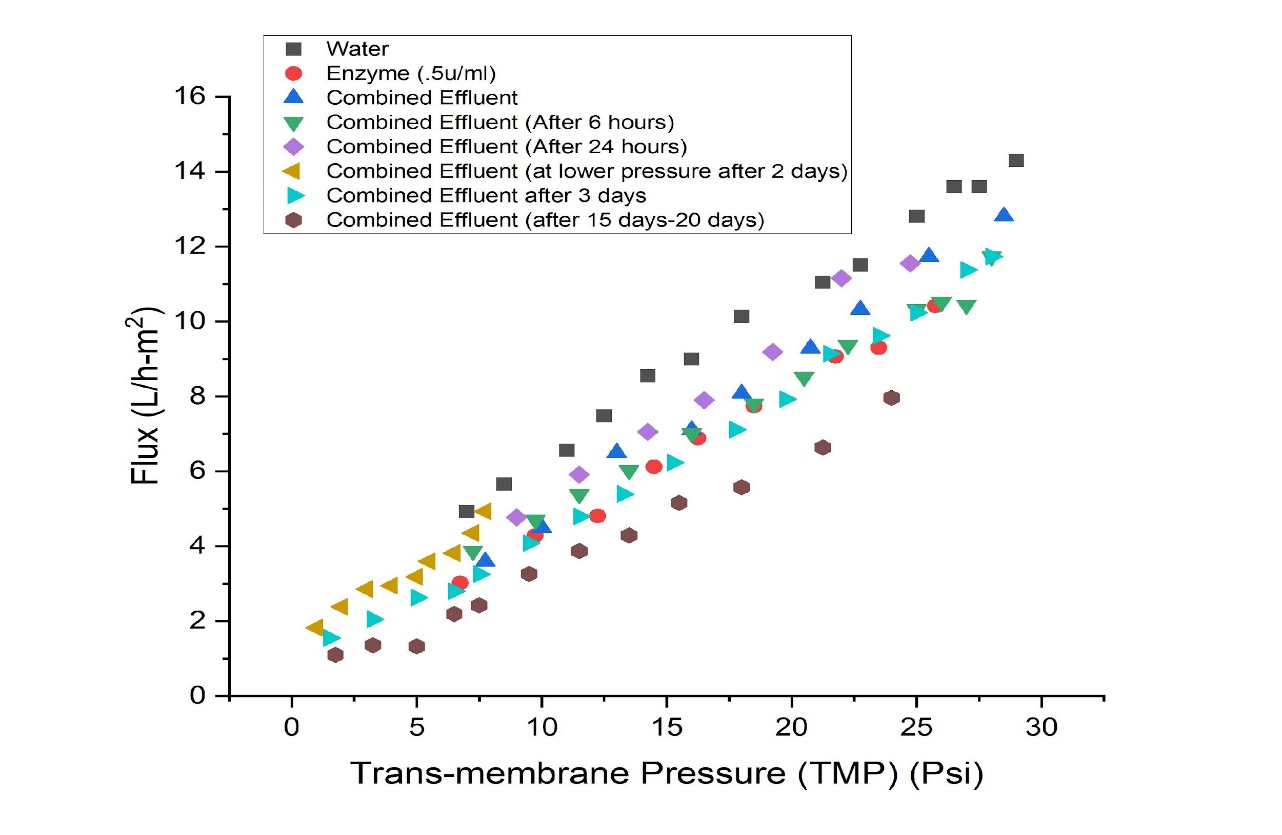


**Supplementary Fig. S5.** Absorption spectrum of the permeate samples collected at regular intervals during the control study performed in the batch reactor. Adsorption of the dye ceased after 20 h of reactor operation. The inset figure shows the percentage increase in color of the permeate samples collected at regular intervals.


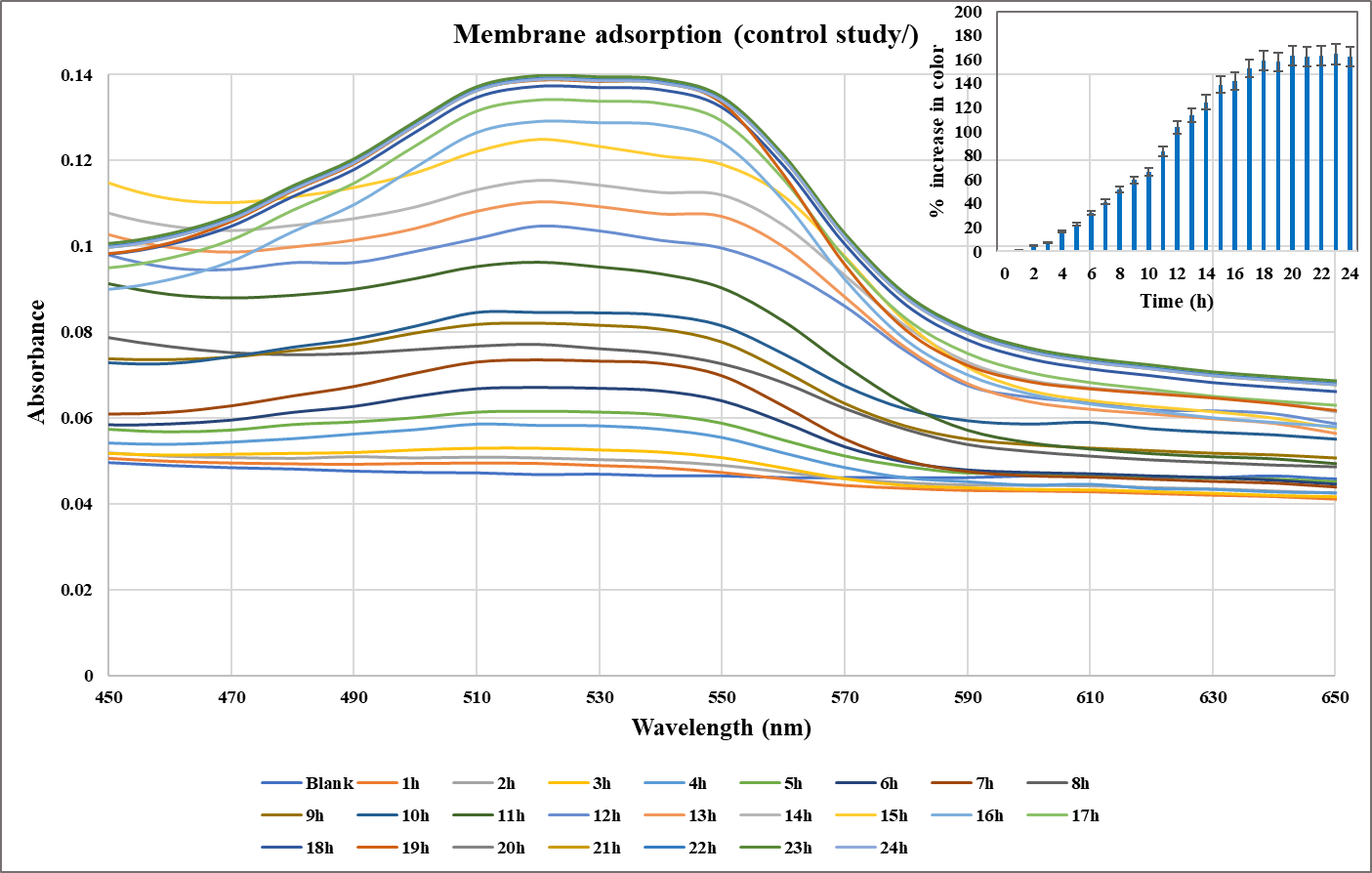


**Supplementary Fig. S6.** Absorption spectrum of the untreated combined effluent, of the permeate after membrane equilibration (without laccase treatment), of the permeate obtained after laccase treatment and plain water, called blank (panel 1) in a batch run. The actual color of the original effluent (panel 2) and the permeate obtained after enzyme treatment (panel 3).


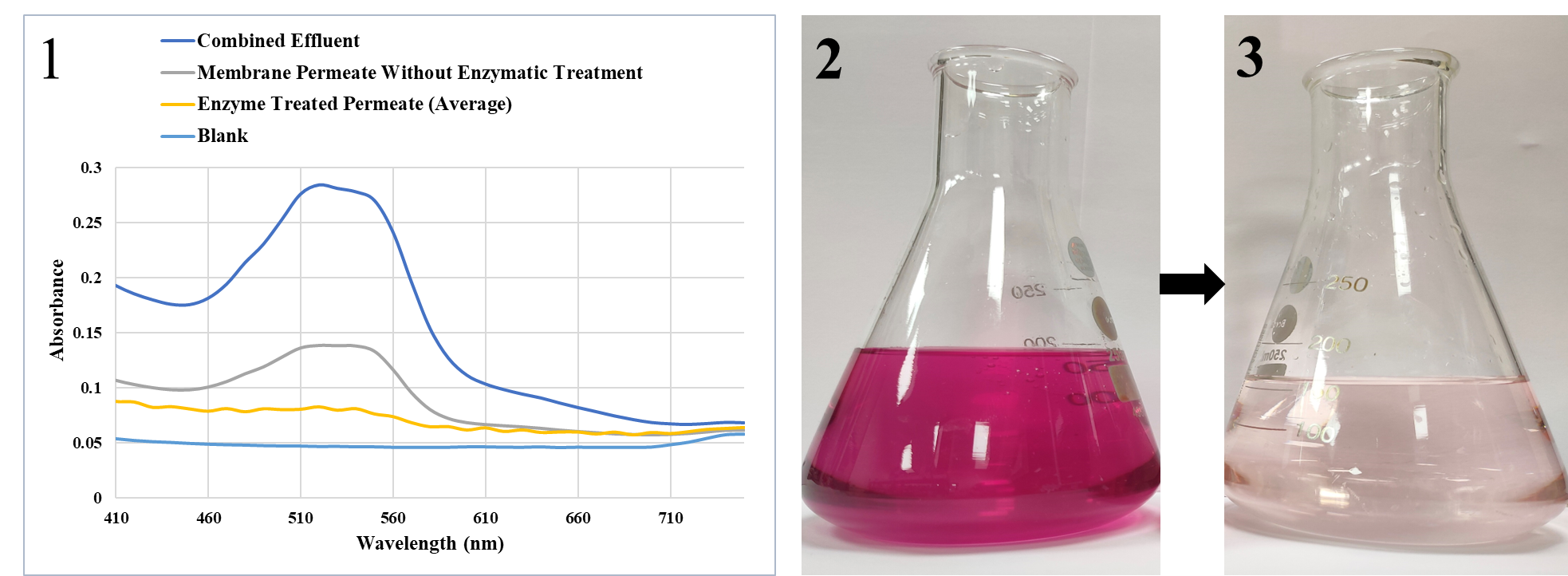

Supplement: Supplementary file 1 [file Data_Sheet_1.docx]
