# Supplementary material for: Detoxification and decolorization of complex textile effluent in an enzyme membrane reactor: batch and continuous studies
Source: Front Microbiol. 2023 Jul 7;14:1193875. doi: 10.3389/fmicb.2023.1193875 (PMC10361525; doi:10.3389/fmicb.2023.1193875)

**Supplementary Fig. S7**. The membrane flux as a function of TMP was recorded before the start and after completion of the reactor run conducted at the HRT of 3 and 6 h.


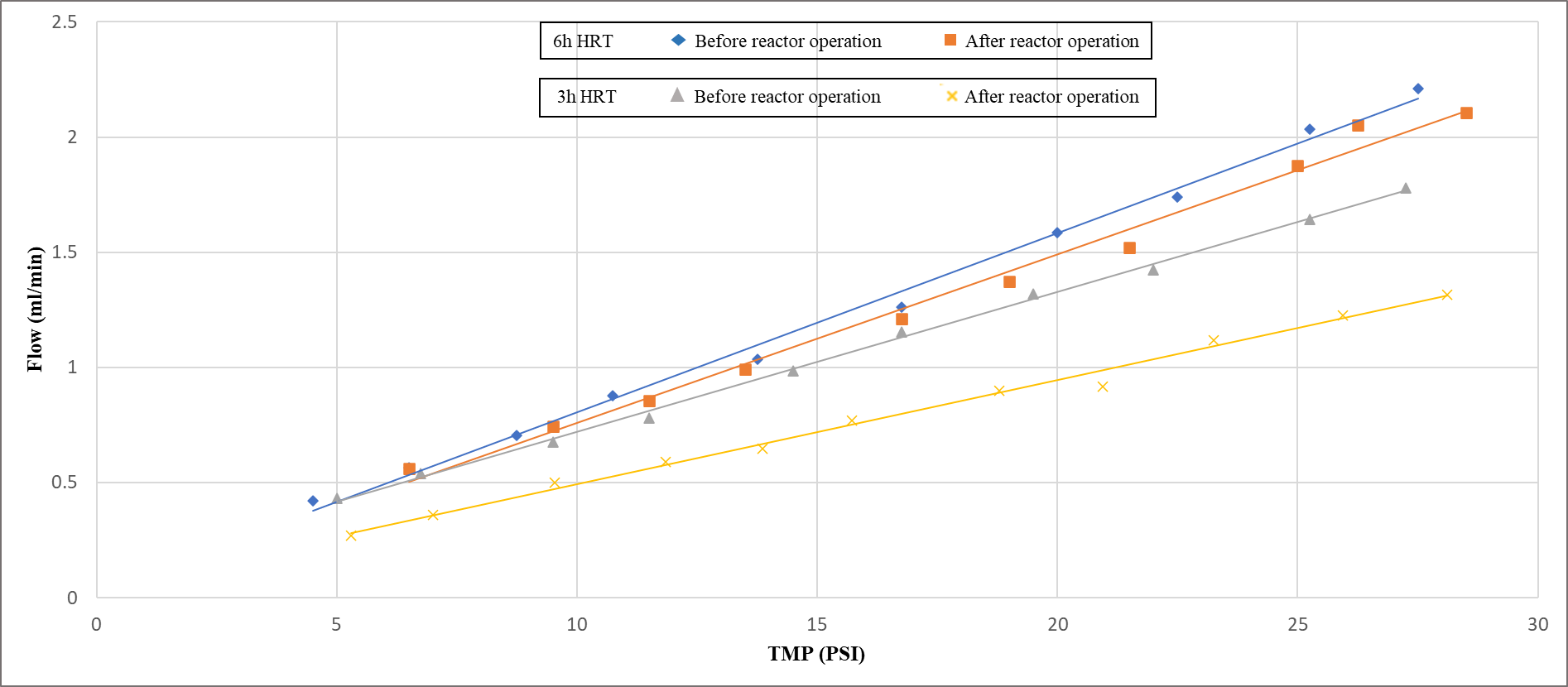

Supplement: Supplementary file 2 [file Data_Sheet_2.docx]
